# Editorial: Family Interventions in Psychosis Change Outcomes in Early Intervention Settings – How Much Does the Evidence Support This?

**DOI:** 10.3389/fpsyg.2018.00406

**Published:** 2018-05-23

**Authors:** Juliana Onwumere, Jens E. Jansen, Elizabeth Kuipers

**Affiliations:** ^1^Institute of Psychiatry, Psychology & Neuroscience, King's College London, London, United Kingdom; ^2^South London and Maudsley NHS Foundation Trust, Bethlem Royal Hospital, Beckenham, United Kingdom; ^3^Psykiatrisk Center København, afd. Rigshospitalet, Region Hovedstadens Psykiatri, Copenhagen, Denmark

**Keywords:** early psychosis, family intervention, carers, schizophrenia, therapy

## Caregiving at the first episode

An episode of psychosis is likely to affect seven percent of the adult population (McGrath et al., 2016) and more than 21 million people worldwide are living with a diagnosis of schizophrenia (WHO, 2017), the most severe form of psychosis. Though, baseline rates of psychosis vary from one region to another, significantly higher rates are consistently reported for young people and black and minority ethnic populations (Jongsma et al., 2017). Decades of research confirm the impact of psychosis extends far beyond the individual who has the diagnosis. It can impact tremendously on the family unit and close social networks of the identified patient. Thus, much has been written of the adverse impact of psychosis and the caregiving role (e.g., burden) on carer well-being and functioning, and the implications for carer and patient outcomes (Kuipers et al., 2010; Gupta et al., 2015; Poon et al., 2016).

The wider literature attests that informal carers of people with psychosis (e.g., parents, siblings, partners, adult offspring), when compared to the general population, report significantly higher rates of common mental disorders and psychological distress (Gupta et al., 2015; Hayes et al., 2015). These rates often reach peak levels during the early illness phases (McCann et al., 2011; Jansen et al., 2015; Sadath et al., 2017). Carers can experience high levels of burden that can include, to varying degrees, reports of loss and grief, trauma, stigma, fatigue, and financial hardship (Patterson et al., 2005; Kuipers et al., 2010; Gupta et al., 2015; Kingston et al., 2016; Onwumere et al., 2017). The constellation of difficulties exists alongside marked social isolation with evidence illustrating psychosis carers are up to 10 times more isolated than non-caregiving peers (Hayes et al., 2015) but also report greater isolation than carers of adults with other health disorders (Magliano et al., 2005).

From its initial onset, the experience of psychosis can be overwhelming and represent a confusing, stress provoking, and life changing experience for relatives (McCann et al., 2011; Lavis et al., 2015). Families can all too often find themselves exposed to a wide range of patient symptoms and behaviors that are difficult to make sense of and equally challenging to cope with (e.g., negative symptoms, auditory hallucinations, paranoid beliefs). Patient behaviors can also include those which are perceived as more anti-social and stigmatizing in presentation such as problematic gambling (Haydock et al., 2015) and aggression (Onwumere et al., 2014). For many relatives, whilst information and support are often needed, the emotional impact of the illness and its effects on their day-to-day functioning can render it difficult for relatives to even be aware of what their information and support needs are (Lavis et al., 2015).

Given the large body evidence detailing the negative impact of psychosis on carer well-being and the influential role that carers can have on patient outcomes, evidence based family interventions are included in treatment guidelines for psychosis across the globe including Europe (National Institute for Health Care Excellence, 2014), America (Kreyenbuhl et al., 2010), Canada (Norman et al., 2017), and Australia (Galletly et al., 2016). In the UK, for example, the current guidelines from the National Institute of Health and Care Excellence (NICE) recommend the provision family based interventions for people living with psychosis in regular contact with families (National Institute for Health Care Excellence, 2014). Iterations of these guidelines have been in existence since the early 2000s (National Institute for Health Care Excellence, 2003, 2009). Moreover, the ongoing development of specialist mental health services for those experiencing their first episode of psychosis also underscores the importance of offering recommended therapeutic interventions for families (Dixon et al., 2015; Marwaha et al., 2016). The UK has witnessed recent developments in the introduction of new access and waiting time standards to ensure that carers and families are accessing the evidence based interventions in a systematic and timely fashion (NHS England, 2016).

## Family interventions at first episode psychosis

For common sense and ethical reasons, few would argue against the need to intervene in early illness phases and offer supportive interventions to families of people experiencing psychosis to improve their outcomes. However, exploring the degree to which the current evidence base provides support for this approach is of importance. In this Research Topic, we saw from the interesting work of Bowman et al. the specific impact psychosis exerts on siblings of first episode psychosis (FEP) relatives and how carer outcomes can be impacted. Drawing on survey data of 157 FEP siblings, the authors highlighted greater levels of carer burden in siblings when there was a history of patient harm to self (i.e., suicide attempts) or violence to others. Moreover, it was younger female siblings who tended to report higher levels of carer burden and be more affected by psychosis in a sibling. Negative caregiving experiences had a significant impact on the overall quality of the caregiving relationship, including the degree of warmth reported between siblings. Rightly, the authors make a call for greater acknowledgment of sibling caregiving roles, which are often be hidden and rarely assessed (Sin et al., 2014), and for recognition of their specific service needs. They also support the development of tailored sibling focused interventions. Though, there has been published work on sibling interventions (Sin et al., 2015), the literature remains sparse documenting its effects.

The Jansen et al. paper in this issue presents the therapeutic rationale and supporting data for integrating carer and family based interventions in psychosis with third wave cognitive behavioral therapies. In a large group of FEP carers (*N* = 101), the authors found that lower levels of psychological flexibility in carers, which reflected a greater tendency to engage in experimental avoidance of thoughts, emotions, and feelings, served as a significant predictor of higher levels of psychological distress. Understanding the factors impacting carer functioning, particularly reports of carer distress and the mechanisms by which they impact, provides greater scope in how we should target evidence based family interventions. To date, our understanding of the exact mechanism through which family interventions yield positive effects for patient and carer groups remains limited. Qualitative data indicate that patient and family groups highlight the importance of psychoeducation, developing a shared understanding of the illness, enhancing skills in problem solving and communication (Nilsen et al., 2016) and having a safe place to discuss issues and learn about their patterns of relating to one another (Rapsey et al., 2015). In addition, reported changes in carer empathy and engagement styles are confirmed mediators of improved outcomes in family based interventions in psychosis (Girón et al., 2015). Consequently, the contribution from Jansen et al. offers encouraging evidence for future testable hypotheses about potential mechanisms that facilitate positive change outcomes in interventions with early psychosis carers.

The robust evidence base in support of the application of family interventions in psychosis continues to develop (Pharoah et al., 2010; Sin et al., 2017). In this topic series, Ruggeri et al.'s paper illustrates the effects of family interventions in psychosis delivered as part of a multi-center cluster trial at first episode. Carers receiving family interventions vs. those in standard care reported significantly greater reductions in subjective levels of caregiver burden, psychological stress, and improved satisfaction with services. The contribution from Claxton et al. compliments the trial data from Ruggeri et al. to attest the efficacy of family interventions in early psychosis groups. Their systematic review of family interventions for early psychosis populations and meta-analysis confirmed the superior effect of the therapy for improving social functioning in patients and reducing rates of relapse. The interventions also yielded beneficial effects for key carer outcomes including burden and well-being. The findings support previous reviews of family interventions in early psychosis (Bird et al., 2010) and a recent systemic review and meta-analysis, which focused specifically on carer outcomes from family interventions in early psychosis (Ma et al., 2017).

Digital applications in healthcare provision are increasingly evident (Cotter et al., 2014; Hollis et al., 2015) and witnessed in mental health conditions (Ebert et al., 2015), including psychosis (Alvarez-Jimenez et al., 2014; Berry et al., 2016). Compared to patient groups, digital applications with family groups have been limited (Rotondi et al., 2005, 2010; Glynn et al., 2010; Onwumere and Kuipers, 2017). The interesting contributions from Chan et al. and Gleeson et al. in the Research Topic renew much needed attention to the potential impact of digital applications for families at first onset and during the early illness phase. Chan et al. reported on findings from an Internet based psychoeducation intervention programme for FEP carers in Hong Kong. It offered different treatment components, including a moderated discussion forum for carers, opportunities for carers to communicate directly with expert clinicians and expert video interviews. Carers provided positive feedback of the intervention and evidence suggested that carers, even at first episode, can engage with an online approach to support their needs. Gleeson et al. describe the background to their online intervention model, which is being tested as part of a cluster RCT with FEP carers. The intervention model combines a therapy component, alongside opportunities for social networking for carers and facilitated support from peers and professionals.

## Conclusion

The papers in this Research Topic speak to the broad evidence base in support of the rationale and delivery of family interventions with early psychosis populations. There is a need to evaluate their application with carer sub groups (e.g., siblings) and when using digital platforms. The contributions also illustrate an importance for further research that seeks to explore and identify key therapy components and mechanisms that yield positive patient and carer outcomes.

## Author contributions

All authors listed have made a substantial, direct and intellectual contribution to the work, and approved it for publication. JO completed the initial draft with EK and JJ making revisions and editing.

### Conflict of interest statement

The authors declare that the research was conducted in the absence of any commercial or financial relationships that could be construed as a potential conflict of interest.
